# Beneficial effects of cocoa, coffee, green tea, and garcinia complex supplement on diet induced obesity in rats

**DOI:** 10.1186/s12906-016-1077-1

**Published:** 2016-03-12

**Authors:** Chi-Chang Huang, Yu-Tang Tung, Wen-Ching Huang, Yi-Ming Chen, Yi-Ju Hsu, Mei-Chich Hsu

**Affiliations:** Graduate Institute of Sports Science, National Taiwan Sport University, Taoyuan, 33301 Taiwan; Graduate Institute of Athletics and Coaching Science, National Taiwan Sport University, Taoyuan, 33301 Taiwan; Department of Sports Medicine, Kaohsiung Medical University, Kaohsiung, 80708 Taiwan

**Keywords:** High energy diet, Polyphenols, Cholesterol, LDL-C, Lipase, Fatty Liver

## Abstract

**Background:**

Cocoa, coffee, green tea and garcinia contain large amounts of polyphenols. Polyphenols are well-known phytochemicals and found in plants, and have modulated physiological and molecular pathways that are involved in energy metabolism, adiposity, and obesity.

**Methods:**

To evaluate the obesity-lowering effect of a combined extract (comprising cocoa, coffee, green tea and garcinia; CCGG) in high-energy diet (HED)-induced obese rats. Male Sprague Dawley rats (8 weeks old) were randomly divided into four groups (*n* = 12 per group): normal diet with vehicle treatment (Control), and HED to receive vehicle or CCGG by oral gavage at 129, 258, or 517 mg/kg/day for 4 weeks, designated the HED, 0.5X, 1X and 1X groups, respectively.

**Results:**

HED induced macrovesicular fat in the liver and the formation of adipose tissues, and significantly increased the levels of serum free fatty acids (FFA), triacylglycerol (TG), total cholesterol (TC), low density lipoprotein cholesterol (LDL-C), and LDL-C/HDL-C, aspartate aminotransferase (AST), alanine aminotransferase (ALT) and ketone bodies in serum, and hepatic TG and TC levels, and decreased the levels of high density lipoprotein cholesterol (HDL-C) in serum and lipase activity in fat tissues. Treatment with CCGG could significantly decrease the levels of FFA, TG, TC, LDL-C, and LDL-C/HDL-C, AST, ALT, and ketone bodies in serum, and hepatic TG and TC contents, and increase the levels of HDL-C in serum and lipase activity in fat tissues compared to the HED group. Liver histopathology also showed that CCGG could significantly reduce the incidence of liver lesions.

**Conclusion:**

These results suggested that CCGG stimulated lipid metabolism in HED-induced obese rats, which is attributable to fat mobilization from adipose tissue.

## Background

Obesity is a serious medical health problem in the world, and was estimated over 500 million adults in 2008 according to the World Health Organization [1]. Obesity, which is an imbalance between energy intake and expenditure, is associated with increased health-care costs, reduced quality of life, and increased risk of various chronic diseases such as heart and cardiovascular disease, type 2 diabetes, hypertension, hypercholesterolemia, and various forms of cancer [2–4]. Genetic, physiological, psychological and gut microbial factors are responsible for the significant increase in prevalence of obesity and its sequels [5–7]. Currently, the available therapeutic approaches for treating obesity have a number of side effects. Therefore, it is an increasing interesting in natural products as anti-obesity agents [8, 9].

Recently, natural bioactive phytochemicals present in natural products have been discovered for their potential health benefit effects on the prevention of chronic disorders such as cancer, cardiovascular disease, inflammatory and metabolic diseases including obesity and insulin resistance. Polyphenols, a class of naturally-occurring phytochemicals, have been shown to modulate physiological and molecular pathways that are involved in energy metabolism, adiposity, and obesity [10]. In cell cultures and animal models of obesity, and in some human clinical and epidemiological studies have demonstrated that polyphenols have beneficial effects on adiposity and obesity as complementary agents in the up-regulation of energy expenditure [10].

Cocoa, coffee, green tea and garcinia (CCGG) all contain large amounts of polyphenols including proanthocyanidins, chlorogenic acids, catechins, and xanthones [11–14]. Among them, there were strong evidences that dyslipidaemia mediated by cocoa, coffee, green tea and garcinia; insulin resistance mediated by cocoa, coffee, and garcinia; inflammation mediated by cocoa, coffee, green tea and garcinia [15–19]. Recently, we demonstrated that a 6-week treatment of CCGG supplementation significantly reduced serum lipid content (TC, TG, and LDL-C) and hepatic lipid content (TC and TG) in high cholesterol diet induced hamsters, and speculated this was attributable to the intake of polyphenols. Such a TG-lowering effect was of interest to us because TG accumulation in liver is one of the major causes of fatty liver diseases [20]. To evaluate the TG-lowering effect of dietary CCGG in obese rats, we fed animals with a moderately high energy diet containing 8 % soybean oil and 44 % sweetened condensed milk and supplemented with three different dose of CCGG for 4 weeks.

## Methods

### Materials

A commercially available supplement consisting of CCGG (beans of *Theobroma cacao*, beans of *Coffea robusta*, leaves of *Camellia sinensis* and fruits of *Garcinia mangostana*) was provided by Sunrider International (CA, USA) and employed as dietary treatment. The CCGG contained 5.2 kcal/g with 30 ‰ (wt/wt) as protein, 290 ‰ (wt/wt) as total fat, 4 ‰ (wt/wt) as saturated fat, 0 ‰ (wt/wt) as trans fat, 610 ‰ (wt/wt) as carbohydrate, 0.05 ‰ (wt/wt) as sodium, 50 ‰ (wt/wt) as dietary fiber, 0.003 ‰ (wt/wt) as iron, and 0.02 ‰ (wt/wt) as calcium. In addition, the total polyphenol and EGCG in CCGG were 196 mg/g and 4.0 mg/g, respectively. The supplement was stored at room temperature and kept in the dark and dry cabinet. The treatment was freshly prepared for daily administration.

### Animals and treatment

**Fig. 1:**
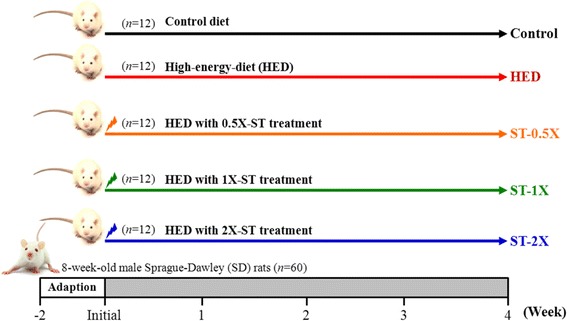
Experimental design. After 2-week adaption, 60 rats were divided randomly into five groups: the normal group was fed a standard chow diet (control, *n* = 12) and the experimental group with a high-energy diet (HED, *n* = 48). The 48 obese rats were divided into four groups (*n* = 12/each group): 1) HED with sedentary control (HED), 2) HED with 129 mg/kg rat/day CCGG (CCGG −0.5X), 3) HED with 258 mg/kg rat/day CCGG (CCGG -1X) and, 4) HED with 517 mg/kg rat/day CCGG (CCGG -2X)

Male Sprague Dawley rats (8 weeks old) with specific pathogen-free conditions were purchased from BioLASCO (A Charles River Licensee Corp., Yi-Lan, Taiwan). The experimental design was depicted as in the Fig. 1. The experimental animals were given 2 weeks to acclimatize to the environment and diet. All animals were fed a chow diet (No. 5001; PMI Nutrition International, Brentwood, MO, USA) and distilled water *ad libitum*, and maintained at a regular cycle (12-h light/dark) at room temperature (23 ± 2 °C) and 50 ~ 60 % humidity. The bedding was changed and cleaned twice per week. All animal experimental protocols were approved by the Institutional Animal Care and Use Committee (IACUC) of National Taiwan Sport University, and the study conformed to the guidelines of the protocol IACUC-10312 approved by the IACUC ethics committee.

All animals were randomly assigned to five groups (12 rats/group). The dose of CCGG for humans is 2.5 g per day. The rat dose (258 mg/kg) was converted from a human equivalent dose (HED) based on body surface area by the following formula from the US Food and Drug Administration: assuming a human weight of 60 kg, the HED for 2500 (mg)/60 (kg) = 41.67 × 6.2 = 258 mg/kg; the conversion coefficient 6.2 was used to account for differences in body surface area between rat and human. Therefore, the CCGG-0.5X and CCGG-2X would be 129 mg/kg and 517 mg/kg rat/day, respectively. CCGG was dissolved in distilled water. The vehicle control and HED groups was performed with the same volume of distilled water or CCGG solution, respectively, equivalent to body weight. The food intake was monitored daily, and BW was recorded weekly. The feed efficiency was calculated as weight gain/food intake × 100 %.

### HED composition

Rats were fed a standard chow diet or a HED as described in our previous study [21]. The standard chow (No. 5001) contained 3.35 kcal/g with 28.5 % as protein, 13.4 % as fat, and 58.1 % as carbohydrates. The HED contained 8 % (wt/wt) soybean oil, 44 % (wt/wt) sweetened condensed milk (Original, Eagle Brand, Nestle) and 48 % (wt/wt) standard chow, for 3.76 kcal/g with 15.5 % as protein, 33.4 % as fat and 51.1 % as carbohydrates.

### Biochemical analysis of serum samples

At the end of experiment, all rats were killed by 95 % CO_2_ asphyxiation, and blood was immediately collected. The serum was prepared by centrifugation at 1500 × *g*, 4 °C for 15 min. Serum free fatty acids (FFA) were measured by use of Siemens Advia 1800 analyzer (Siemens Healthcare Diagnostics, IL, USA). Serum total cholesterol (TC), low density lipoprotein cholesterol (LDL-C), high density lipoprotein cholesterol (HDL-C) levels were determining by use of Hitachi C1600 analyzer (Hitachi C1600, Hitachi, Japan). The concentrations of serum triacylglycerol (TG), glucose, aspartate transaminase (AST), alanine transaminase (ALT), uric acid (UA), creatinine (CREA), blood urea nitrogen (BUN), ketone bodies, sodium (Na), and potassium (K) were analyzed using Beckman DX 800 (Beckman Coulter, Inc., Fullerton, CA).

### Liver lipid content

After blood collection, liver tissues were excised from rats. To determine liver cholesterol and triglycerides, 20 mg of liver tissue was homogenized in a 200 μL solvent (chloroform : isopropanol : NP40 = 7 : 11 : 0.1). Centrifuged at 12 000 × *g* for 10 min, an aliquot of 100 μL was extracted and dried. The pellet was reconstituted with a buffer (1 M of potassium phosphate, pH = 7.4, 500 mM of sodium chloride, 50 mM of cholic acid), and water bath sonication was employed to dissolve the precipitate. A cholesterol fluorometric assay kit (Cayman, Ann Arbor, MI, USA) and triglyceride colorimetric assay kit (Cayman, Ann Arbor, MI, USA) were used to analyze liver cholesterol and triglyceride contents.

### Lipase activity of epididymal fat pad

The epididymal fat pad (EFP) was excised from rats. To determine lipase activity, 100 mg of EFP was homogenized, centrifuged at 12 000 × *g* for 10 min. and the supernatant was measured using the Bioviaion kit (Biovision, Milpitas, CA, USA) in accordance with the manufacturer’s specifications.

### Histological staining of tissues

Liver, fat, kidney, and heart tissues were collected and immediately fixed in 10 % formalin after being weighed. Tissue was then embedded in paraffin and cut into 4-μm thick slices, then stained with hematoxylin and eosin (H&E) and examined under a light microscope equipped with a CCD camera (BX-51, Olympus, Tokyo) by a clinical pathologist.

### Statistical analysis

All data were expressed as mean ± SD. Statistical differences were analyzed by one-way ANOVA with Duncan’s test. Mann–Whitney U-test was only used for data comparisons of ketone bodies. *P* < 0.05 was considered statistically significant.

## Results

### Effects of CCGG supplementation on body weight, diet intake, and food efficiency

**Fig. 2:**
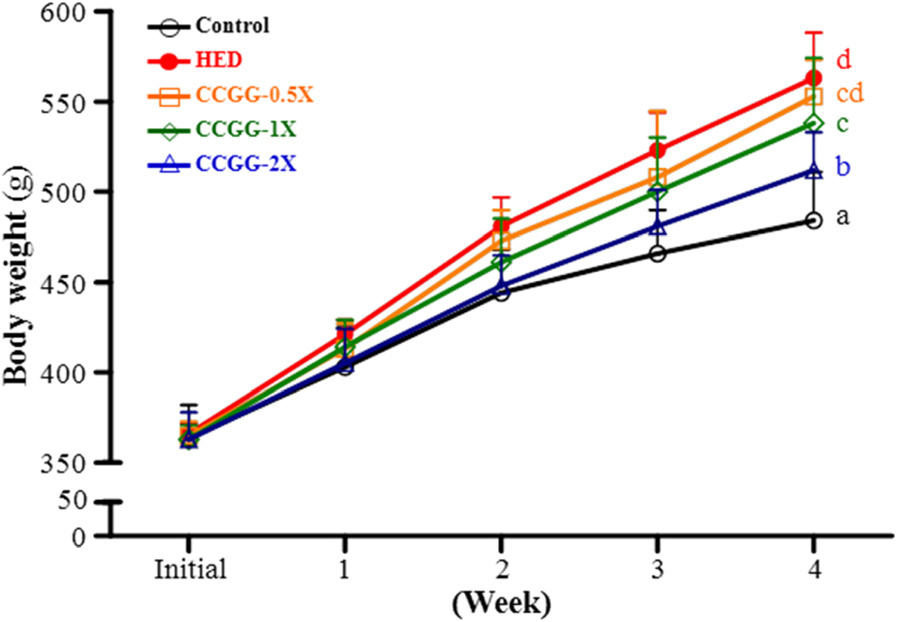
Body weights over the course of 4 weeks. Control; vehicle control, HED; high-energy diet control, 0.5X; HED with 129 mg/kg/d of CCGG, 1X; HED with 258 mg/kg/d of CCGG, 2X; HED with 517 mg/kg/d of CCGG. Data are mean ± SD (*n* = 12 rats/group). Values with different letters (a, b, c, d) differ significantly at *P* < 0.05 by one-way ANOVA

The effects of CCGG supplementation on final body weight, diet intake, and food efficiency in HED-fed rats were shown in Table 1. The initial body weight for control and HED rats were similar. Rats fed on the normal diet and HED continued to show increased body weight (Fig. 2) and food intake until the end of the study. After 4-week induced obesity, the body weight was higher for HED than control rats (*P* < 0.05). After CCGG treatment in the HED rats, the body weight significantly decreased in CCGG-1X and CCGG-2X groups compared to HED group (*P* < 0.05). The average final body weight of CCGG-0.5X, CCGG-1X, and CCGG-2X groups decreased by 2.0, 5.0, and 8.9 % compared with HED group. In addition, the HED groups with or without treatment of CCGG did not cause diarrhea during the experiment. Treatment with HED, CCGG-0.5X, CCGG-1X, and CCGG-2X slightly reduced the daily food intake compared to vehicle group, which is due to HED had higher energy intake than the normal diet. Food efficiency was increased in the HED group compared with the vehicle group, but treatment of CCGG-0.5X, CCGG-1X, and CCGG-2Xsignificantl reduced that value in the HED group groups.

**Table 1:** The effects of CCGG supplementation on final body weight, diet intake, and food efficiency in HED-fed rats

	Control	HED	0.5X	1X	2X
Initial BW (g)	364 ± 18	366 ± 6	365 ± 8	363 ± 8	363 ± 15
Final BW (g)	484 ± 27^a^	563 ± 25^d^	553 ± 20^cd^	538 ± 36^c^	512 ± 21^b^
Diet intake (g/rat/day)	15.9 ± 0.3^c^	15.4 ± 0.3^b^	15.2 ± 0.5^b^	15.3 ± 0.5^b^	14.1 ± 0.3^a^
Feed efficiency (%)	26.9 ± 3.1^a^	45.8 ± 4.2^d^	44.1 ± 3.2^cd^	41.1 ± 6.9^bc^	37.7 ± 3.0^b^

Control, vehicle control; HED, high-energy diet control; 0.5X, HED with 129 mg/kg/d of CCGG; 1X, HED with 258 mg/kg/d of CCGG; 2X, HED with 517 mg/kg/d of CCGG. Data are mean ± SD (*n* = 12 rats/group). Values with different letters (a, b, c, d) differ significantly at *P* < 0.05 by one-way ANOVA

### Effects of CCGG supplementation on fat pads

Fat tissue weights at the end of the study were shown in Table 2. The epididymal fat pad (EFP), renal fat pad (RFP), mesenteric fat pad (MFP) are white adipose tissues in the body. EFP, RFP, and MFP mass was greater for HED-alone than control rats, and CCGG-0.5X, CCGG-1X, and CCGG-2X groups decreased the mass. The relative weight of EFP, RFP and MFP was higher for HED-alone than control rats by 65.5 % (*P* = 0.0092), 354.4 % (*P* < 0.0001), and 103.5 % (*P* < 0.0001), respectively. However, CCGG-0.5X, CCGG-1X, and CCGG-2X groups could decrease the relative weight as compared with HED alone, by 6.4, 5.9 or 18.2 % (*P* = 0.0277), 59.3 % (*P* < 0.0001), 56.0 % (*P* < 0.0001), or 65.3 % (*P* < 0.0001) and 11.6, 12.7, or 19.7 % (*P* = 0.0035), respectively. The body fat percentage of control, HED, CCGG-0.5X, CCGG-1X, and CCGG-2X groups were 2.31 ± 0.37, 5.10 ± 0.92, 3.89 ± 0.78, 3.92 ± 1.07, 3.44 ± 0.74 %, respectively. In addition, CCGG dose-dependently decreased the relative weight of MFP. The body fat percentage of the HED rats was dramatically elevated 120.8 % compared to vehicle rats (*P* < 0.0001). After the CCGG-0.5X, CCGG-1X, and CCGG-2X supplementation were markedly decreased 23.7 % (*P* = 0.0006), 23.1 % (*P* = 0.0008), and 32.5 % (*P* < 0.0001) compared to the HED rats without treatment. Recent studies showed that feeding rats with a high-fat diet induced the changes in several enzymatic reactions related to carbohydrate metabolism in liver and adipose tissue [22, 23]. Thus, it supported the possibility that CCGG may have an alternation on several enzymatic reactions and reduce the formation of adipose tissue.

**Table 2:** The effects of CCGG supplementation on the mass of epididymal fat pad (EFP), renal fat pad (RFP), mesenteric fat pad (MFP), and total body fat in HED-fed rats

	Control	HED	0.5X	1X	2X
EFP (g)	5.24 ± 1.33^a^	10.06 ± 1.94^c^	9.23 ± 2.13^bc^	9.05 ± 3.01^bc^	7.51 ± 2.02^b^
RFP (g)	1.53 ± 0.48^a^	8.09 ± 2.87^c^	3.24 ± 0.91^b^	3.43 ± 1.90^b^	2.54 ± 0.78^ab^
MFP (g)	3.93 ± 0.69^a^	9.33 ± 1.54^c^	8.05 ± 1.91^bc^	7.76 ± 2.16^b^	6.79 ± 1.66^b^
Total body fat (g)	10.7 ± 2.2^a^	27.5 ± 5.8^c^	20.5 ± 4.3^b^	20.2 ± 6.9^b^	16.8 ± 4.0^b^
Relative EFP (%)	1.13 ± 0.23^a^	1.87 ± 1.30^c^	1.75 ± 0.39^bc^	1.76 ± 0.47^bc^	1.53 ± 0.39^b^
Relative RFP (%)	0.33 ± 0.10^a^	1.50 ± 0.49^c^	0.61 ± 0.17^b^	0.66 ± 0.32^b^	0.52 ± 0.15^ab^
Relative MFP (%)	0.85 ± 0.11^a^	1.73 ± 0.24^c^	1.53 ± 0.35^bc^	1.51 ± 0.32^bc^	1.39 ± 0.31^b^
Body fat percentage (%)	2.31 ± 0.37^a^	5.10 ± 0.92^c^	3.89 ± 0.78^b^	3.92 ± 1.07^b^	3.44 ± 0.74^b^

Control, vehicle control; HED, high-energy diet control; 0.5X, HED with 129 mg/kg/d of CCGG; 1X, HED with 258 mg/kg/d of CCGG; 2X, HED with 517 mg/kg/d of CCGG. Data are mean ± SD (*n* = 12 rats/group). Values with different letters (a, b, c) differ significantly at *P* < 0.05 by one-way ANOVA

### Effects of CCGG supplementation on serum biochemical parameters associated with lipid profiles

**Fig. 3:**
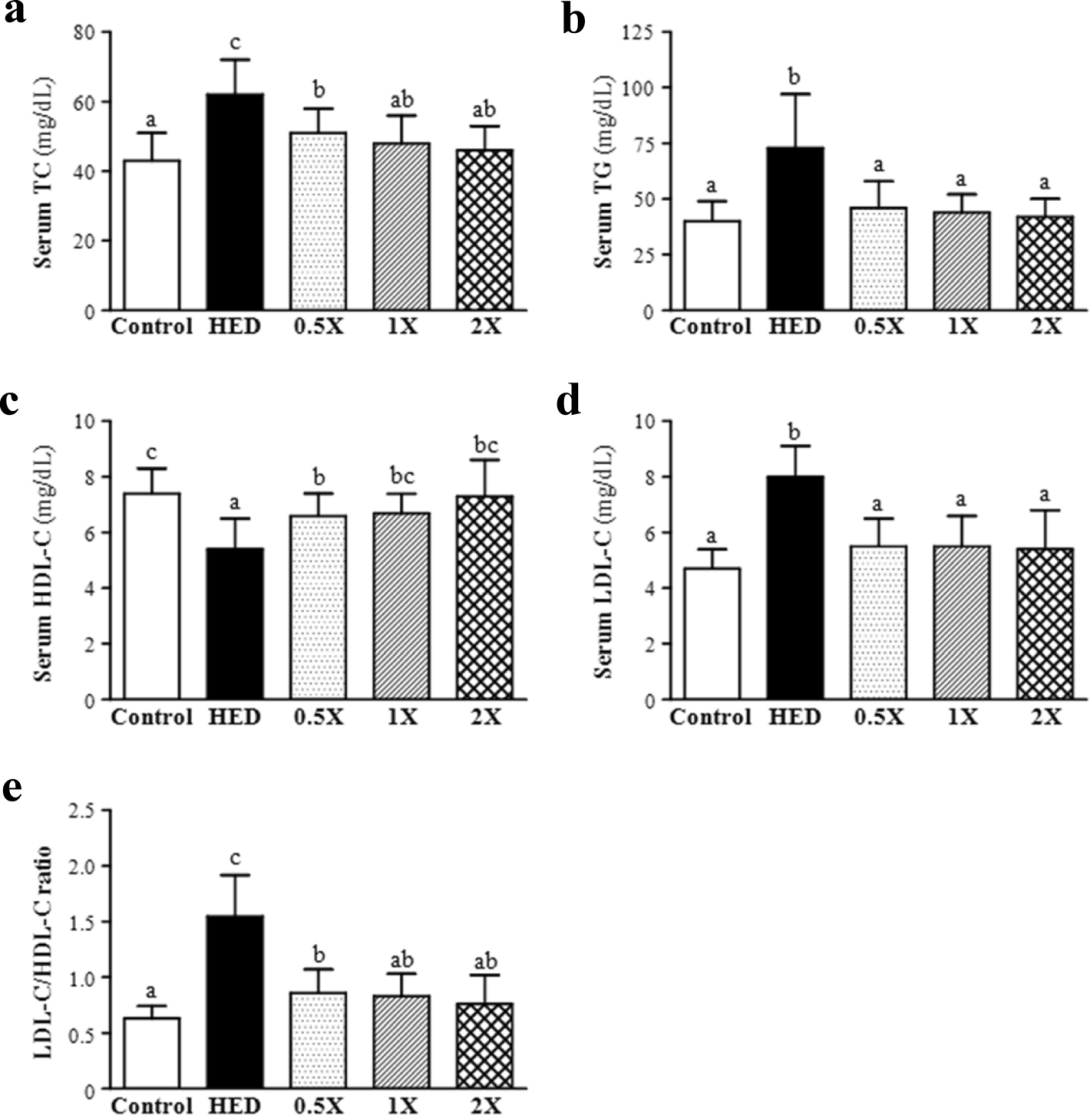
Effects of CCGG supplementation on serum lipids. **a** serum total cholesterol levels. **b** serum triglycerides levels. **c** serum high-density lipoprotein cholesterol. **d** serum low-density lipoprotein cholesterol. **e** serum low-density lipoprotein cholesterol. Control; vehicle control, HED; high-energy diet control, 0.5X; HED with 129 mg/kg/d of CCGG, 1X; HED with 258 mg/kg/d of CCGG, 2X; HED with 517 mg/kg/d of CCGG. Data are mean ± SD (*n* = 12 rats/group). Values with different letters (a, b, c) differ significantly at *P* < 0.05 by one-way ANOVA

Effects of CCGG supplementation on FFA, TG, TC, LDL-C, HDL-C, and LDL-C/HDL-C were shown in Fig. 3. An HED diet fed to the SD rats significantly increased the serum levels of FFA, TG, TC, LDL-C, and LDL-C/HDL-C by 28.6 % (*P* < 0.0001), 82.5 % (*P* < 0.0001), 44.2 % (*P* < 0.0001), 70.2 % (*P* < 0.0001), and 146.0 % (*P* < 0.0001), respectively, and decreased the serum level of HDL-C by 27.0 % (*P* < 0.0001) compared to those in the vehicle group) (Table 3). After the CCGG supplementation, the treated groups exhibited a significant decrease in the serum levels of FFA (12.9, 15.2, and 25.2 % reduction in the CCGG-0.5X, CCGG-1X, and CCGG-2X groups, respectively), TG (30.7, 40.3, and 42.5 % reduction in the CCGG-0.5X, CCGG-1X, and CCGG-2X groups, respectively), TC (18.0, 22.4, and 25.7 % reduction in the CCGG-0.5X, CCGG-1X, and CCGG-2X groups, respectively), LDL-C (30.5, 30.7, and 32.8 % reduction in the CCGG-0.5X, CCGG-1X, and CCGG-2X groups, respectively), HDL-C (22.9, 25.7, and 35.9 % increase in the CCGG-0.5X, CCGG-1X, and CCGG-2X groups, respectively), and LDL-C/HDL-C ratio (44.6, 46.1, and 50.7 % reduction in the CCGG-0.5X, CCGG-1X, and CCGG-2X groups, respectively), compared with the HED group. Strongly dose dependent effects were observed in the serum levels of FFA, TG, TC, HDL-C, and LDL-C/HDL-C.

**Table 3:** The effects of CCGG supplementation on serum biochemical parameters in HED-fed rats

	Control	HED	0.5X	1X	2X
TG (mg/dL)	40 ± 9^a^	73 ± 24^b^	46 ± 12^a^	44 ± 8^a^	42 ± 8^a^
FFA (mmol/L)	1.05 ± 0.14^ab^	1.35 ± 0.06^d^	1.18 ± 0.11^c^	1.15 ± 0.12^bc^	1.01 ± 0.15^a^
TC (mg/dL)	43 ± 8^a^	62 ± 10^c^	51 ± 7^b^	48 ± 8^ab^	46 ± 7^ab^
LDL-C (mg/dL)	4.7 ± 0.7^a^	8.0 ± 1.1^b^	5.5 ± 1.0^a^	5.5 ± 1.1^a^	5.4 ± 1.4^a^
HDL-C (mg/dL)	7.4 ± 0.9^c^	5.4 ± 1.1^a^	6.6 ± 0.8^b^	6.7 ± 0.7^bc^	7.3 ± 1.3^bc^
LDL-C/HDL-C	0.63 ± 0.11^a^	1.55 ± 0.37^c^	0.86 ± 0.21^b^	0.83 ± 0.20^ab^	0.76 ± 0.26^ab^
UA (mg/dL)	1.04 ± 0.22^a^	1.08 ± 0.18^a^	1.03 ± 0.20^a^	1.03 ± 0.17^a^	0.98 ± 0.23^a^
CREA (mg/dL)	0.39 ± 0.03^a^	0.40 ± 0.05^a^	0.39 ± 0.03^a^	0.39 ± 0.05^a^	0.38 ± 0.05^a^
BUN (mg/dL)	8.20 ± 0.75^a^	8.68 ± 1.26^a^	8.08 ± 1.50^a^	8.00 ± 0.93^a^	8.03 ± 1.23^a^
Na (mmol/L)	141 ± 2^a^	140 ± 1^a^	141 ± 1^a^	141 ± 2^a^	141 ± 1^a^
K (mmol/L)	4.3 ± 0.3^a^	4.2 ± 0.3^a^	4.3 ± 0.3^a^	4.3 ± 0.2^a^	4.2 ± 0.4^a^
Ketone bodies	0/12	12/12^*^	8/12^*,†^	3/12^†^	1/12^†^

Control, vehicle control; HED, high-energy diet control; 0.5X, HED with 129 mg/kg/d of CCGG; 1X, HED with 258 mg/kg/d of CCGG; 2X, HED with 517 mg/kg/d of CCGG. Data are mean ± SD (*n* = 12 rats/group). Values with different letters (a, b, c) differ significantly at *P* < 0.05 by one-way ANOVA. Ketone bodies = the rats of the positive ketone bodies/the total rats. *, *P* < 0.05 compared with control group, and †, *P* < 0.05 compared with HED group by Mann–Whitney U-test

### Effect of CCGG supplementation on hepatic triglycerides and total cholesterol

**Fig. 4:**
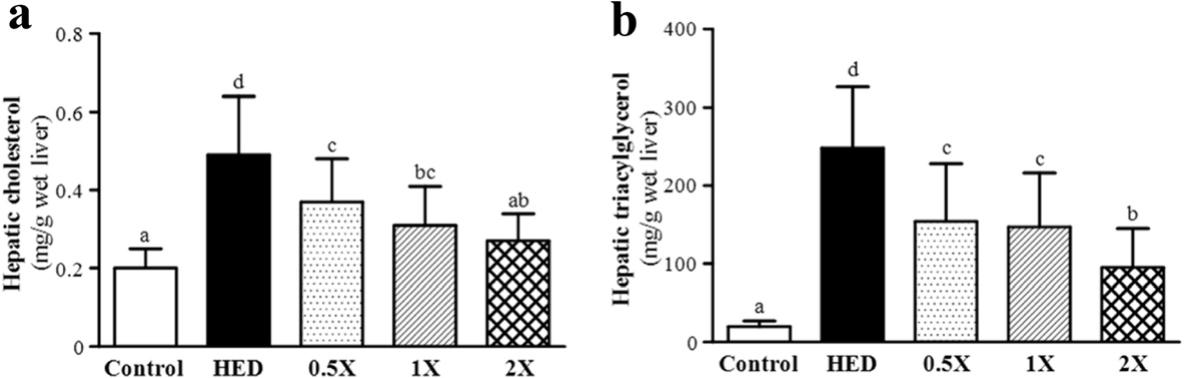
Effects of CCGG supplementation on hepatic lipids. **a**; hepatic total cholesterol levels. **b**; hepatic triglycerides levels. Control; vehicle control, HED; high-energy diet control, 0.5X; HED with 129 mg/kg/d of CCGG, 1X; HED with 258 mg/kg/d of CCGG, 2X; HED with 517 mg/kg/d of CCGG. Data are mean ± SD (*n* = 12 rats/group). Values with different letters (a, b, c, d) differ significantly at *P* < 0.05 by one-way ANOVA

The effects of CCGG supplementation on hepatic TG and TC in HED-fed rats were shown in Fig. 4. An HED diet fed to rats markedly accumulated hepatic TG (a 12.5-fold increase compared with vehicle group, *P* < 0.0001) and hepatic TC (a 2.5-fold increase compared with vehicle group, *P* < 0.0001). In the liver tissues, CCGG-0.5X, CCGG-1X, and CCGG-2X reduced TG (154 ± 74, 147 ± 69, and 95 ± 50 mg/g, respectively) and TC (0.37 ± 0.11, 0.31 ± 0.10, and 0.27 ± 0.07 mg/g) levels compared with the levels of TG and TC (248 ± 78 and 0.49 ± 0.15 mg/g, respectively) in the HED group. Thus, treating HED rats with CCGG-0.5X, CCGG-1X, or CCGG-2X significantly reduced hepatic TG and TC (37.9, 40.7, or 61.6 % and 24.1, 36.8, or 45.0 %, respectively) compared with those in vehicle group. These results supposed that CCGG may have an inhibitory action on cholesterol synthesis in liver, a facilitating effect on biliary excretion of TG.

### Effect of CCGG supplementation on serum biochemical parameters associated with liver function

**Fig. 5:**
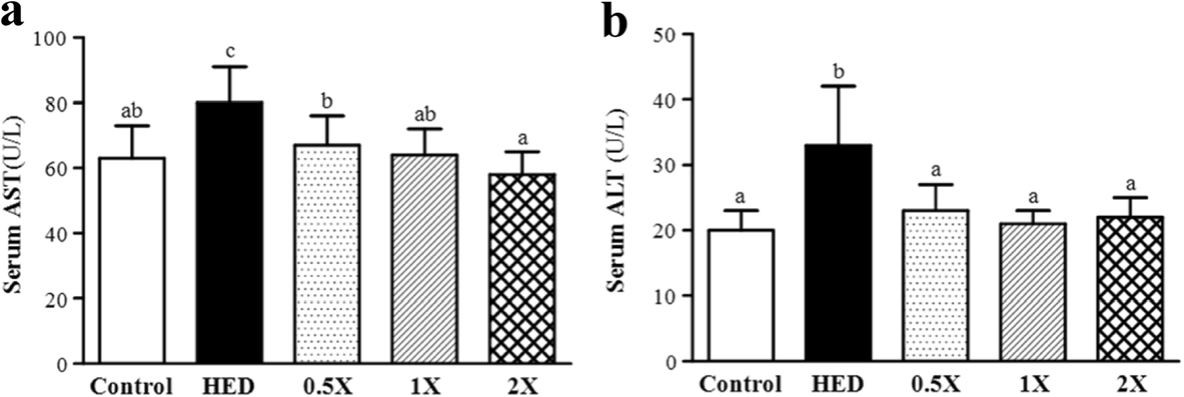
Effects of CCGG supplementation on liver function. **a**; aspartate aminotransferase. **b**; alanine aminotransferase. Control; vehicle control, HED; high-energy diet control, 0.5X; HED with 129 mg/kg/d of CCGG, 1X; HED with 258 mg/kg/d of CCGG, 2X; HED with 517 mg/kg/d of CCGG. Data are mean ± SD (*n* = 12 rats/group). Values with different letters (a, b, c) differ significantly at *P* < 0.05 by one-way ANOVA

AST and ALT have long been considered effective indicators of hepatic injury. Effect of CCGG supplementation on serum AST and ALT levels were shown in Fig. 5. The serum AST and ALT levels of the HED rats were dramatically elevated (*P* < 0.0001) to 80 ± 11 and 33 ± 9 U/L, respectively, compared to those of rats in the vehicle group, which were 63 ± 10 and 20 ± 3 U/L, respectively. The HED rats treated with CCGG-0.5X, CCGG-1X, or CCGG-2X exhibited significantly lower levels of serum AST and ALT (67 ± 9, 64 ± 8, or 58 ± 7 and 23 ± 4, 21 ± 2, or 22 ± 3 U/L, respectively), compared with the HED group (*P* < 0.0001).

### Effect of CCGG supplementation on serum biochemical parameters

Effect of CCGG supplementation on serum biochemical parameters were shown in Table 3. As indicators of nephrotoxicity, UA, CREA, and BUN showed no differences among all groups (*P* > 0.05). In addition, it appeared that all groups have no different serum electrolyte levels including sodium (Na^+^) and potassium (K^+^) (*P* > 0.05). Thus, CCGG did not cause toxicology during the experiment. Glucose also showed no differences among all groups (*P* > 0.05). Chang et al. [24] showed that a high-fat diet slightly increased glucose level compared to the vehicle group, but the difference was not statistically significant. Our result was a similar to their report. On the other hand, an HED diet fed to the SD rats produced the ketone bodies (100 %; 12/12) compared with vehicle rats (0 %; 0/12) (*P* < 0.05), which may be due to ketone bodies are produced by the liver from fatty acids. Treatment with CCGG-0.5X (67 %; 8/12), CCGG-1X (25 %; 3/12), or CCGG-2X (8 %; 1/12) significantly decreased the ketone bodies compared with the HED group (*P* < 0.05).

### Effect of CCGG supplementation on lipase activity

The lipoprotein lipase (LPL) activity of control, HED, CCGG-0.5X, CCGG-1X, and CCGG-2X groups were 1.09 ± 0.43, 0.28 ± 0.13, 0.50 ± 0.20, 0.57 ± 0.14, 0.61 ± 0.17 mU/mL, respectively. LPL activity of the HED rats was dramatically decreased 74.4 % compared to vehicle rats (*P* < 0.0001). After the CCGG-0.5X, CCGG-1X, and CCGG-2X supplementation were markedly elevated 1.78- (*P* = 0.0298), 2.05- (*P* = 0.0040), and 2.20-fold (*P* = 0.0012) compared to the HED rats without treatment. It is well known that the LPL is important in the metabolism of lipoproteins and also in the regulation of plasma HDL levels [25–31]. Numerous studies showed that subjects with high plasma LPL activity have high HDL levels [25–27], and these results were a similar to this study that CCGG-0.5X, CCGG-1X, and CCGG-2X supplementation with high serum LPL activity have high HDL levels.

### Effect of CCGG supplementation on hepatomegaly

**Fig. 6:**
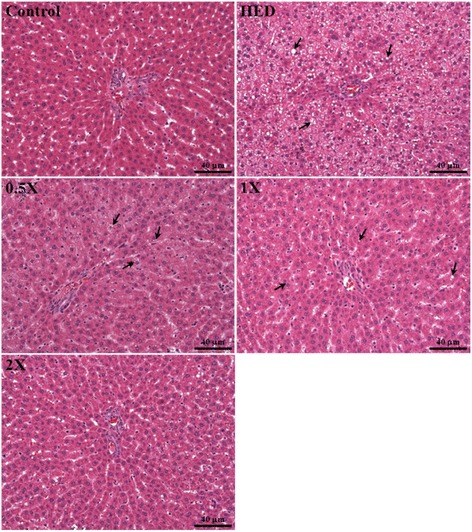
Histopathology of liver tissues. (H & E stain, magnification: 200×, Scale bar: 40 μm). Control; vehicle control, HED; high-energy diet control, 0.5X; HED with 129 mg/kg/d of CCGG, 1X; HED with 258 mg/kg/d of CCGG, 2X; HED with 517 mg/kg/d of CCGG

Rats fed with high-fat diet for 4 weeks developed a higher degree of lipid deposition of liver tissues. Livers of the HED rats were grossly larger compared with those in the vehicle group and were pale in color, and their adipose tissues were also markedly increased. This higher fatty content (Fig. 4b) was confirmed by H & E staining of the liver sections, and severe cytoplasmic vacuoles in the hepatocytes were observed in the HED group, whereas the histology appeared normal in the vehicle group (Fig. 6). The fatty liver was represented as mixed (macro- and microvesicular) lipid droplets in the HED group. Feeding with HED also resulted in a more prominent inflammation and necrosis which was corroborated by morphological analysis of the abundance of inflammatory cells and pericentral necrosis. In addition, livers of the HED group showed widespread deposition of lipid droplets of different sizes inside the parenchymal cells (arrow position, Fig. 6). In contrast, livers from the vehicle group showed scattered, negligible droplets of fat which were not visibly. Treatment with CCGG-0.5X, CCGG-1X, and CCGG-2X reduced hepatomegaly and the formation of fatty liver and fat in these rats. These results indicate that treating HED rats for 4 weeks with CCGG-0.5X, CCGG-1X, and CCGG-2X decreased the accumulation of macrovesicular fat in the liver and the formation of adipose tissues. In addition, H&E staining of the kidneys and hearts revealed showed no differences among all groups (data not shown).

## Discussion

Obesity causes the accumulation of excess TG in adipose tissue and leads to adverse health problems, including heart disease, cerebrovascular disease, diabetes, hypertension, hypercholesterolemia and other chronic diseases. [1]. Although the etiology of obesity is complex, dietary factors, particularly the consumption of HED, is considered a risk factor for its development. The numerous studies indicated that consumption of HED leads to obesity and then rats fed HED weighed more than normal diet rats and they developed substantially more adipose tissue than normal rats and acquired the hyperlipidemia effects typically associated with obesity [32]. The HED-induced rat has been widely used as a model for the evaluation of hyperlipidemic prevention and has many similarities between the metabolism diseases observed in HED-induced rats and in humans. The purpose of this study was to systematically investigate the effects of CCGG on HED-induced obesity in rats.

Cocoa, coffee, green tea and garcinia (CCGG) contain abundant polyphenols, particularly proanthocyanidins, chlorogenic acids, catechins and xanthones, respectively [11–14]. In recent years, there has been increasing interest in polyphenols from a wide variety of traditional herbal for against blood lipids and related inflammatory responses plants [33–36]. Xu et al. [37] revealed that polysaccharides markedly reduced the leptin expressions in white adipose tissue, and serum leptin levels in obese patients were significantly higher than the normal group.

In this study, cumulative food intake over the experimental period was similar in both groups. Rats fed with high-fat diet for 4 weeks markedly increased body weight compared to normal diet group. This showed that the HED group had higher energy intake compared to the normal diet group, and the HED contributed to migration of obesity. These results were a similar to these studies [38–40]. Parallel to the change of body weight, the weights of white adipose tissues (EFP, RFP, and MFP) were higher in the HED group than in the normal diet group. Treating HED rats with CCGG-0.5X, CCGG-1X, and CCGG-2X for 4 weeks significantly reduced body weight gain, and the attenuated body weight of the treated animals may be due to the presence of a decreased amount of fatty tissue and a decreased adipocyte size. The histological appearance and size of white adipocytes in HED groups were drastically larger than the vehicle group but those of CCGG-0.5X, CCGG-1X, and CCGG-2X groups were quite smaller than HED alone group. In addition, HED diet fed to the SD rats significantly increased the serum levels of FFA, TG, TC, LDL-C, LDL-C/HDL-C AST and ALT, and decreased the serum level of HDL-C compared to those in the vehicle group. After the CCGG supplementation, the treated groups exhibited a significant reduction in the serum levels of FFA, TG, TC, LDL-C, LDL-C/HDL-C AST and ALT, and increasing in the serum level of HDL-C compared to those in the vehicle group. Shepherd and Kahn [41] showed that obese and diabetic patients increased plasma FFA levels, which is due to abnormal release by insulin-resistant adipocytes. With insulin resistance, the combination of elevated plasma concentrations of fatty acids promotes hepatic fatty acid synthesis and impairs β-oxidation, which further leads to hepatic steatosis [42]. Thus, treatment with CCGG-0.5X, CCGG-1X, or CCGG-2X may decrease hepatic fatty acid synthesis recovery β-oxidation, which further reduced hepatic steatosis.

CCGG contains cocoa, coffee, green tea and garcinia. Nwichi et al. [16] reported that an 8-weeks administration of cocoa extract significantly reduced hyperlipidaemia in cholesterol-fed rats. Gu et al. [15] found that 8 % unsweetened cocoa powder (approximately equivalent to 465 mg/kg/d in human) for 10-weeks significantly ameliorated obesity-related inflammation, insulin resistance, and fatty liver disease. Song et al. [17] found that that an 11-weeks administration of 0.3 % and 0.9 % decaffeinated green coffee bean significantly reduced high-fat-diet induced body weight gain and increments in plasma lipids, glucose, and insulin levels. Panchal et al. [43] showed that 5 % (approximately equivalent to 294 mg/kg/d in human) aqueous extract of coffee for 8 weeks significantly attenuated hypertension and impairment in glucose homeostasis without affecting abdominal fat deposition and plasma lipid profile on a rat model of human metabolic syndrome. Xu et al. [37] demonstrated that 400 or 800 mg/kg/d green tea polysaccharides and polyphenols treated with high-fat diet fed rats for 6 weeks significantly reduce serum lipids. Luo et al. [18] demonstrated that supplementation with 1.0 or 2.0 g/kg body weight (approximately equivalent to 161 or 323 mg/kg/d in human) green tea leaf powder for 30 days resulted in a significant decrease in plasma TC levels and circulating immune complexes and an increase in HDL-C. Dulloo et al. [44] also found that administration of a green tea extract significantly increased energy expenditure and fat oxidation in a group of young males. In addition, several clinical trials have reported the effects of tea preparations on increasing energy expenditure, fat oxidation, weight loss, fat mass, and weight maintenance after weight loss [44–47]. Bumrungpert et al. [19] revealed the garcinia reduced inflammation and insulin resistance in human adipocytes. Chae et al. [48] have showed that 200 mg/kg BW (approximately equivalent to a dose of 16.26 mg/kg/d for humans) of *Garcinia mangostana* extract for significantly decreased TC, TG, and LDL-C levels in mice. Chang et al. [24] demonstrated that a 6-week treatment of CCGG supplementation significantly reduced serum lipid content (TC, TG, and LDL-C) and hepatic lipid content (TC and TG). Concerning our results showed effective dose started at CCGG-0.5X group (equivalent to 21 mg/kg/d in human). Therefore, compared to previous studies focusing on single ingredient, the CCGG might have the synergic beneficial effects with relatively lower dose consumption.

## Conclusion

We demonstrated hypolipidemic effects of a 6-weeks dietary supplementation using a combined extract (consisting of CCGG) in HED-fed SD rats. CCGG markedly attenuated serum and hepatic lipid profiles with dose-dependent effects, and increased HDL-C. It is therefore suggested that CCGG may represent a new type of hypolipidemic agents and they may have a potent effects of ameliorating hyperlipidemia, insulin resistance, liver steatosis, and related inflammation.

### Availability of data and materials

Data are all contained within the paper.
